# Canadian Pregnancy Outcomes in Rheumatoid Arthritis and Systemic Lupus Erythematosus

**DOI:** 10.1155/2011/345727

**Published:** 2011-10-19

**Authors:** Cheryl Barnabe, Peter D. Faris, Hude Quan

**Affiliations:** ^1^Department of Medicine, University of Calgary, Calgary, AB, Canada T2N 4N1; ^2^Alberta Bone & Joint Health Institute, Calgary, AB, Canada T2N 4Z6; ^3^Department of Community Health Sciences, University of Calgary, Calgary, AB, Canada T2N 4N1

## Abstract

*Objective*. To describe obstetrical and neonatal outcomes in Canadian women with rheumatoid arthritis (RA) or systemic lupus erythematosus (SLE). *Methods*. An administrative database of hospitalizations for neonatal delivery (1998–2009) from Calgary, Alberta was searched to identify women with RA (38 pregnancies) or SLE (95 pregnancies), and women from the general population matched on maternal age and year of delivery (150 and 375 pregnancies, resp.). Conditional logistic regression was used to calculate odds ratios (OR) for maternal and neonatal outcomes, adjusting for parity. *Results*. Women with SLE had increased odds for preeclampsia or eclampsia (SLE OR 2.16 (95% CI 1.10–4.21; *P* = 0.024); RA OR 2.33 (95% CI 0.76–7.14; *P* = 0.138)). Women with SLE had increased odds for cesarean section after adjustment for dysfunctional labour, instrumentation and previous cesarean section (OR 3.47 (95% CI 1.67–7.22; *P* < 0.001)). Neonates born to women with SLE had increased odds of prematurity (SLE OR 6.17 (95% CI 3.28–11.58; *P* < 0.001); RA OR 2.66 (95% CI 0.90–7.84; *P* = 0.076)) and of SGA (SLE OR 2.54 (95% CI 1.42–4.55; *P* = 0.002); RA OR 2.18 (95% CI 0.84–5.66; *P* = 0.108)) after adjusting for maternal hypertension. There was no excess risk of congenital defects in neonates. *Conclusions*. There is increased obstetrical and neonatal morbidity in Canadian women with RA or SLE.

## 1. Introduction

Historically, epidemiology studies have documented deleterious effects of rheumatic disease on pregnancy [1]. Over the last fifteen years, the approach to treating rheumatic diseases has changed substantially, with a focus on aggressive treatment to achieve remission, and an increased selection of immunosuppressive therapies. Preconception and prenatal care have received increased attention as these newer therapies have become available, requiring additional patient counseling on potential teratogenicity [2]. It is worthwhile to examine what impact these changes may have had on obstetrical and neonatal outcomes in women with rheumatic disease.

In fact, recent evidence from large administrative databases from the United States, Taiwan, Norway, Denmark, and Sweden is in keeping with the historical literature, with demonstrated discrepancies in obstetrical and neonatal outcomes for rheumatology patients relative to the general population. At the population level, women with rheumatoid arthritis (RA) or systemic lupus erythematosus (SLE) experience more preeclampsia [3–8] and have a higher risk of cesarean section [3–7]. Their neonates are at increased risk of being small for gestational age (SGA) and premature [3–7, 9]. There may be increased perinatal mortality [7, 9]. 

 We are unaware of any contemporary data on obstetrical or neonatal outcomes in Canadian patients with rheumatic disease; therefore, we used population-based data from a Canadian urban centre to determine obstetrical outcomes in women with RA or SLE over the last decade, relative to those in women of the general population. Maternal outcomes of interest included the frequency of preeclampsia or eclampsia, cesarean section, the occurrence of postpartum infections, and the effect of these complications on the length of stay and the need for intensive care unit admission. We also examined neonatal outcomes, including the frequency of prematurity and congenital defects, gestational size, and the need for special care unit admission. An estimation of the risks attendant to childbearing will allow rheumatologists and obstetrical care providers to improve the prenatal information that is provided to patients with rheumatic disease.

## 2. Methods

### 2.1. Data Source

The study population was determined from a population-based hospital discharge abstract database for the Calgary Zone of Alberta Health Services (with a population of 1.3 million people and three tertiary care hospitals), for fiscal years 1998/1999 to 2008/2009, after ethical approval from the University of Calgary Health Research Ethics Board. Prior to 2002, coding was performed using the International Classification of Diseases, Ninth Revision, Clinical Modification (ICD-9-CM) for diagnosis and procedures. Thereafter the International Classification of Diseases, Tenth Revision, Canadian Enhancement (ICD-10-CA), and the Canadian Classification of Health Interventions (CCI) for diagnoses and procedures were used, respectively. Standard admission and discharge information for all hospital separations using 16 (ICD-9-CM) and 25 (ICD-10-CA) diagnostic and 10 procedure code fields are captured. This same data is used by the Canadian Institute for Health Information in their surveillance activities, and coders receive significant training and are audited to ensure accuracy. The database exists primarily for administrative purposes within the context of provincial health care delivery and therefore does not include information on an individual's rheumatic disease characteristics such as duration, severity, or treatment.

### 2.2. Study Population

Women between the ages of 12 and 55 years who had a hospital separation for delivery of a neonate (gestational age >20 weeks) and with diagnostic codes for RA (ICD-9-CM 714.0; ICD-10-CA M05 or M06) or SLE (ICD-9-CM 710.0; ICD-10-CA M32) were identified from the database. The general population comparison group consisted of women with an obstetrical admission randomly selected from the same administrative database, in a 4 : 1 ratio, matched on maternal age (within two years) and year of delivery (same calendar year). Some women with rheumatic disease had more than one delivery during the study period, and each delivery was matched to a new control. The neonates were identified by their unique personal health numbers recorded in a field of the mother's chart. Only singleton births were included in the analysis. Advanced maternal age was considered to be ≥36 years of age.

### 2.3. Outcomes

Diagnostic and procedure codes were used to identify outcomes and covariates. The maternal outcomes of interest for this study were (1) maternal preeclampsia or eclampsia (including worsening of preexisting hypertension during pregnancy) (ICD-9-CM 642.x; ICD-10-CA O10-O16), (2) cesarean section (ICD-9-CM procedures 74.x; ICD-10-CA procedures 5MD60), and (3) postpartum infection (ICD-9-CM 670.0, 658.4, or 674.3; ICD-10-CA O85 and O86). Neonatal outcomes of interest were (1) congenital defects (ICD-9-CM 740–759; ICD-10-CA Q00-Q99), (2) prematurity, and (3) gestational size. The Public Health Agency of Canada defines preterm births as those occurring before 37 weeks of gestation and SGA as babies who were less than the 10th percentile for weight based on their gestational age, weight, and sex [10]. We also determined the length of stay and intensive care requirements for both mothers and neonates. We included information on readmissions within a one-year time period for both the mother and the neonate to identify delayed events.

### 2.4. Explanatory Variables

Parity (first birth, ≥second birth) was included in all models. Additional covariates for cesarean section were dysfunctional labour, instrumentation to assist delivery, and previous cesarean section. Additional covariates for postpartum infection were instrumentation and cesarean section. Neonatal outcomes were also adjusted for maternal hypertension (preexisting cases and new diagnoses).

### 2.5. Statistical Methods

Conditional logistic regression was used to calculate crude odds ratios (OR) and their 95% confidence intervals (CI) for the outcomes of interest. An adjusted OR was calculated, taking into account specific confounders for each outcome that were available as described above. Due to the sample size retrieved from our search, we limited the number of explanatory variables in our analysis. The predetermined alpha level for statistical significance was set at 5% (two tailed). Statistical analysis was performed using STATA IC 10.0 (StataCorp, College Station, Texas) and R version 2.13.0 (available at http://www.r-project.org/).

## 3. Results

We matched 38 singleton births in women with RA and 95 singleton births in women with SLE to 150 and 375 singleton births in women of the general population, respectively. The mean age of women with RA was 32 years (standard deviation (SD) 5.5, range 21–42) and with SLE was 30 years (SD 5.5, range 20–45). Twenty-nine percent (29%) of women with RA and 13% of women with SLE were of advanced maternal age. The median parity for women with RA or SLE was 0, compared to a median parity of 1 for women of the general population.

Maternal outcomes are summarized in Table 1. Women with RA or SLE had a longer mean length of stay in hospital (mean difference RA: 0.8 days; mean difference SLE: 1.8 days). Preeclampsia or eclampsia occurred in 18.4% of pregnancies of women with RA and 16.8% of pregnancies of women with SLE, compared to 7.3% and 9.3% of their controls, respectively. A large proportion of women with RA or SLE delivered by cesarean section (RA: 34.2% versus 21.3%; SLE: 43.2% versus 23.7%). Women with SLE experienced a higher frequency of postpartum infections. These infections were either of the uterine cavity (*n* = 2), the surgical wound (*n* = 2), or were coded as puerperal sepsis with no location specified (*n* = 2). In two SLE patients, maternal infection was associated with stillbirth of an extremely premature baby. All other infections were associated with either cesarean section or instrumentation for delivery. 

In the first postpartum year, more women with RA or SLE were readmitted compared to their controls. Two of these readmissions were specifically for SLE flares. The median time to readmission was similar between women with rheumatic disease (80 days, interquartile range (IQR) 6–288) and their controls (94 days, IQR 9–225). No women required intensive care during either the index admission or during a readmission.

Neonatal outcomes are summarized in Table 2. Although the mean number of gestational weeks was similar between babies born to mothers with RA or SLE and their controls (RA: mean 38.1 (SD 2.0) versus 36.2 (SD 10.0) weeks; SLE: mean 37.2 (SD 3.4) versus 37.5 (SD 7.6) weeks), a higher proportion of the neonates of mothers with rheumatic disease were born prematurely. These neonates more frequently required intensive care support. Intubation was required in 5.3% of neonates born to mothers with RA compared to 4.7% for their controls and 4.2% of neonates born to mothers with SLE compared to 1.9% for their controls. There were three stillbirths in the SLE group compared to two in the control group, but specific details for the causes of death were not available.

Babies born to mothers with rheumatic disease were smaller, with 28.9% of RA babies (versus 12.0%) and 25.3% of SLE babies (versus 10.9%) meeting the criteria for SGA. The difference in birth weight between babies born to mothers with rheumatic disease and their controls was more pronounced in SLE. The mean birth weight difference between RA neonates and their controls was 298 grams for females (95% CI 10–586; *P *= 0.0425) and 388 grams for males (95% CI 102–675; *P *= 0.0084), compared to 533 grams (95% CI 344–722; *P* < 0.001) for female SLE neonates and 512 (95% CI 310–713; *P* < 0.001) for male SLE neonates. Neonates of women with rheumatic disease were not at increased risk of readmission. There was also no evidence of excess congenital defects in babies born to women with rheumatic disease. 

Controlling for parity, interesting trends emerged for obstetrical and neonatal outcomes in RA or SLE. Women with rheumatic disease remain at substantial risk for preeclampsia or eclampsia compared to the general population (SLE OR 2.16 (95% CI 1.10–4.21; *P* = 0.024); RA OR 2.33 (95% CI 0.76–7.14; *P* = 0.138)) (Table 3). Women with SLE were also more likely to deliver by cesarean section (adjusted OR 3.47 (95% CI 1.67–7.22; *P* < 0.001)) compared to their controls, after additionally adjusting for dysfunctional labor, instrumentation, and previous cesarean section. The possible increased risk of postpartum infections in women with SLE was negated once adjustments for instrumentation at delivery and cesarean sections were made. Neonates of women with rheumatic disease were more likely to be preterm compared to general population controls after additionally adjusting for maternal hypertension (SLE adjusted OR 6.17 (95% CI 3.28–11.58; *P* < 0.001); RA adjusted OR 2.66 (95% CI 0.90–7.84; *P* = 0.076)). These neonates were also more likely to meet SGA criteria (SLE adjusted OR 2.54 (95% CI 1.42–4.55; *P* = 0.002); RA adjusted OR 2.18 (95% CI 0.84–5.66; *P* = 0.108)).

## 4. Discussion

Our results demonstrate that women with RA or SLE, in a Canadian urban setting with universal health coverage, have adverse obstetrical outcomes. They have at least twice the odds of developing preeclampsia or eclampsia relative to the general population, and a large proportion deliver by cesarean section. Neonates of women with RA or SLE are at least three times as likely to be premature and small for gestational age. These neonates require admission to the intensive care unit for additional supportive care and therefore may be at increased risk for long-term morbidity. Table 3 summarizes the results of our study compared to those reported in other countries.

A few prospective cohorts have examined pregnancy outcomes in SLE and inflammatory arthritis. A prospective Canadian SLE cohort found that women with active renal disease, compared to those without active renal disease, more frequently had pregnancy-induced hypertension and disease flare, with lower birth weight babies and more congenital malformations [11]. Two prospective cohorts of women with inflammatory polyarthritis and RA found that higher disease activity during pregnancy resulted in babies of lower birth weight [12, 13]. 

The biologic mechanisms responsible for differential pregnancy outcomes have not been determined [14]. Pregnancy in normal individuals is associated with a shift from a Th1 cytokine milieu to a Th2 predominant one. Women with RA have been shown to have the same response, which may account for remission of disease observed during pregnancy. However, endocrine effects and other potential immunomodulatory mechanisms have not been adequately studied. Inflammation likely plays a role, as increased disease activity during pregnancy has resulted in babies of lower birth weight, as demonstrated in the prospective RA cohorts [12, 13]. 

Our study is limited by the inability to adjust for important disease-related confounders for the women with RA or SLE, such as disease duration, disease activity, or medication exposures in the pregestational or gestational period. We also are unable to comment on an individual's characteristics which may affect pregnancy outcomes, such as smoking status, body mass index, ethnicity, or the quality or quantity of prenatal care received for each individual. These variables are not available in the discharge abstract database. Our analysis considered each individual birth occurring in a woman with RA or SLE, with new controls selected each time, in order to optimize the size of our dataset, the resulting effect estimates, and their precision. We adjusted for parity (first birth or ≥second birth) rather than restricting the analysis to first birth only such as was done in the studies by Wallenius et al. [7] and Nørgaard et al. [9] due to the limited number of births to women with RA or SLE in our dataset. 

Our use of conditional logistic regression did not allow us to account for outcomes in mothers with more than one birth during the study period. In the SLE group fourteen mothers had two births, and three mothers in the RA group had two births. The associations among outcomes for these mothers may have resulted in confidence intervals that were narrower than warranted by the data. To test for the impact of obstetrical outcomes for those with more than one birth in the study period, we attempted to fit a mixed-effects model with random effects for pair and mother. However, these models did not converge, possibly due to the small number of mothers with more than one birth during the study period and also because those with more than one birth during the study period were only in the RA or SLE group. As a sensitivity analysis, we also fitted GEE logistic regression models that conditioned on the matching variables and used a working correlation matrix to account for mothers with more than one birth. The inclusion of the working correlation matrix had negligible impact on the confidence intervals and *P* values (data available upon request).

Our findings are not at particular risk for selection bias, as the universal Canadian health care system ensures equal access to hospital care, and all admissions are captured. A potential source of bias may have been related to errors in coding, with the potential for misclassification of either the outcome or the disease status determination [15]. Patients with mild rheumatic disease that did not complicate the pregnancy or hospital course could have been missed in the coding process. The effect of this error could falsely increase the event rate in the control group, as women with RA or SLE would erroneously be included here. Determination of outcome events would depend on the accuracy of the chart and data extraction by the coder. Coders are trained and audited regularly, so the potential source of this error is small and would affect both case and control groups equally, thus maintaining the effect size between the two groups. 

We propose the following explanations to explain why there is ongoing discrepancy in obstetrical outcomes for rheumatic disease patients in the modern treatment era. It is possible that improved treatment approaches have had little or no impact on the underlying biologic mechanisms responsible for adverse obstetrical outcomes, in particular for women who have had long-standing disease. Women planning a pregnancy may be unwilling to be exposed to newer agents or aggressive management for fear of teratogenic effects. Women with more severe disease, who may have previously been unable to conceive, may now achieve pregnancy due to aggressive immunosuppressive treatment and may be at higher risk for obstetrical and neonatal complications. Reproductive technologies are also increasingly available, affecting the risk of complications as well. It is also possible that treatment is preventing even more deleterious outcomes from occurring that cannot be measured, as that would be counterfactual. 

In summary, our study confirms that women with RA or SLE remain at increased odds of developing preeclampsia or eclampsia, undergoing a cesarean section, and a trend to developing more postpartum infections. Their neonates are more frequently born preterm, are of lower weight, and more frequently require intensive care. These risks can be communicated to patients with rheumatic disease and highlight the need for collaborative care between rheumatologists and prenatal care providers.

## Figures and Tables

**Table 1:** Summary of maternal outcomes.

Outcome	Women with RA *n *= 38	RA controls *n* = 150	Women with SLE *n* = 95	SLE controls *n* = 375
Length of stay (days), mean (SD)	3.0 (1.5)	2.2 (1.2)	4.2 (5.3)	2.3 (2.4)
Preeclampsia, *n *(%)				
Mild	7 (18.4)	11 (7.3)	9 (9.5)	33 (8.8)
Severe/eclampsia	0 (0.0)	0 (0.0)	7 (7.4)	2 (0.5)
Cesarean section, *n* (%)	13 (34.2)	32 (21.3)	41 (43.2)	89 (23.7)
Postpartum infection, *n* (%)	0 (0.0)	1 (0.7)	6 (6.3)	5 (1.3)
1 year readmission, *n* (%)	7 (15.8)	6 (4.7)	13 (13.7)	22 (5.9)

*n:* number; SD: standard deviation.

**Table 2:** Summary of neonatal outcomes.

Outcome	Neonates of women with RA *n* = 38	Neonates of RA controls *n* = 150	Neonates of women with SLE *n* = 95	Neonates of SLE controls *n* = 375
Prematurity, *n* (%)				
<28 weeks gestation	0 (0.0)	0 (0.0)	3 (3.2)	0 (0.0)
28–34 weeks gestation	1 (2.6)	1 (0.7)	8 (8.4)	7 (1.9)
34–37 weeks gestation	7 (18.4)	12 (8.0)	22 (23.2)	19 (5.1)
Requiring intensive care, *n* (%)	11 (28.9)	17 (11.3)	34 (35.8)	50 (13.3)
Small for gestational age, *n* (%)	11 (28.9)	18 (12.0)	24 (25.3)	41 (10.9)
Congenital defects*, *n* (%)	0 (0.0)	3 (2.0)	2 (2.1)	6 (1.6)
1 year readmission, *n* (%)	2 (5.3)	8 (5.3)	10 (10.5)	18 (4.8)

*n*: number; SD: standard deviation.

*Includes chromosomal abnormalities, cleft lip and/or palate, and talipes calcaneovalgus.

**Table 3:** Local and worldwide published odds ratios (and 95% confidence intervals) for obstetrical and neonatal complications.

Publication and country	Disease	Pregnancy-related hypertension*	Cesarean section	Prematurity (<37 weeks gestation)^†^	Size^‡^	Postpartum infection
Barnabe, Canada	RA	2.33 (0.76–7.14)	1.89 (0.53–6.78)^§^	2.66 (0.90–7.84)^||^	2.18 (0.84–5.66)^||^	NR
	SLE	2.16 (1.10–4.21)	3.47 (1.67–7.22)^§^	6.17 (3.28–11.58)^||^	2.54 (1.42–4.55)^||^	2.07 (0.50–8.54)^¶^
Chakravarty, United States [3]	RA	1.4 (1.0–2.0)	1.5 (1.2–1.9)	NR	2.3 (1.2–4.3)	NR
	SLE	3.4 (2.9–4.1)	1.6 (1.4–1.9)	NR	3.7 (2.7–5.2)	NR
Clowse, United States [4]**	SLE	3.0 (2.7–3.3)	1.7 (1.6–1.9)	2.4 (2.1–2.6)	2.6 (2.2–3.1)	Sepsis: 3.5 (2.0–6.0) Pneumonia: 4.3 (3.1–5.9)
Reed, United States [5]^††^	RA	1.55 (0.97–2.50)	1.66 (1.22–2.26)	1.78 (1.21–2.60)	1.51 (0.94–2.43)	NR
Lin, Taiwan [6]	RA	2.23 (1.60–3.12)	1.20 (1.09–1.33)	1.18 (0.98–1.40)	1.20 (1.05–1.37)	NR
Wallenius, Norway [7]	Chronic inflammatory arthritides	0.60 (0.22–1.62)	1.71 (1.13–2.59)	1.91 (1.13–3.24)	1.59 (0.99–2.54)	NR
Nørgaard, Denmark and Sweden [9]	RA	1.50 (1.13–1.94)	1.78 (1.56–2.02)	1.53 (1.23–1.90)	1.60 (1.25–2.03)	NR
Wolfberg, United States [8]^‡‡^	RA	1.8 (0.2–13.4)	NR	NR	NR	NR
	SLE	5.7 (2.0–16.2)	NR	NR	NR	NR

All values are crude odds ratios (95% confidence intervals) unless otherwise stated.

RA: rheumatoid arthritis; SLE: systemic lupus erythematosus; OR odds ratio; CI: confidence interval; SGA: small for gestational age; IUGR: intrauterine growth restriction; NR: not reported.

*All pregnancy-related hypertensive disorders. In the studies by Clowse, Reed, Lin, Wallenius, Nørgaard, and Wolfberg odds ratios for preeclampsia are reported.

^†^Clowse reports preterm labor. Nørgaard reports prematurity as 32–36 weeks gestation.

^‡^All report small for gestational age, except Chakravarty and Clowse which report intrauterine growth restriction.

^§^Adjusted for dysfunctional labor (including uterine inertia, malpresentation, or obstruction) and previous cesarean section.

^||^Adjusted for instrumentation and caesarean section.

^¶^Adjusted for maternal hypertension.

**Adjusted for comorbidities, pregnancy complications, and medical computations.

^††^Relative risk calculated from odds ratio; adjusted for age, smoking, year. ^‡‡^Adjusted for age.
